# Combined surgery with intramedullary rod fixation across the ankle for the treatment of Crawford IV congenital pseudarthrosis of the tibia: a long-term follow-up study

**DOI:** 10.1186/s13023-025-03873-3

**Published:** 2025-07-04

**Authors:** Xiping Liu, Kun Liu, Guanghui Zhu

**Affiliations:** Department of Orthopedics, Hunan Children’s Hospital, NO.86 Ziyuan Road, Yuhua District, Changsha City, 410007 Hunan Province China

**Keywords:** Congenital pseudarthrosis of the tibia, Combined surgery, Refracture

## Abstract

**Background:**

The combined surgery with tibial intramedullary (IM) rod fixation across the ankle for the treatment of Crawford IV congenital pseudarthrosis of the tibia (CPT) is the most classic and effective surgical treatment method. The aim of this study is to explore the long-term clinical efficacy of combined surgery for the treatment of Crawford IV congenital pseudarthrosis of the tibia (CPT).

**Methods:**

A retrospective analysis was conducted on 82 cases of Crawford IV CPT that underwent combined surgical treatment with tibial IM rod fixation through the ankle, to evaluate their long-term clinical efficacy and postoperative complications.

**Results:**

The average surgical age of the patient was (43.17 ± 13.40) months (range: 18–96 months), with an initial bone healing rate of 100%, and an average postoperative follow-up time of (119.78 ± 20.08) months (range: 84–146 months); At the last follow-up, there were a total of 20 cases of re fractures, including 15 cases in the complete insertion of IM rods into the tibial medullary cavity group, 2 cases in the tibial IM rod across the ankle fixation group, and 3 cases in the tibial IM rod removal group; There were a total of 28 cases of developmental bending and angular deformity of the tibial shaft after bone healing, including 26 cases in the complete insertion of IM rods into the tibial medullary cavity group, 0 cases in the tibial IM rod across the ankle fixation group, and 2 cases in the tibial IM rod removal group; All cases in the complete insertion of IM rods into the tibial medullary cavity group showed tibial IM rods deviating from the center of the medullary cavity, with a displacement rate of up to 100%.

**Conclusions:**

The combined surgery of tibial IM rod fixation across the ankle for the treatment of Crawford IV CPT has a high initial bone healing rate and definite therapeutic effect, but there are still many postoperative complications. The fixation status of tibial IM rods is an important influencing factor for tibial shaft developmental angular bending deformity and re fractures after initial bone healing.

## Background

Congenital pseudarthrosis of the tibia (CPT) is a rare disease that is among the most difficult conditions to cure in pediatric orthopedics, and its incidence rate is 1/250,000 ~ 1/140,000 [1]. Multiple classification systems have been proposed to describe the classification changes of this disease, among which Crawford classification is the most commonly used method, and the anterior tibial arch is a common feature of its four types. Type I: increased and widened cortical density of the tibia; Type II: narrowing of the tibial medullary cavity with sclerosis; Type III: presence of cysts or pre fracture signs; Type IV: presence of pseudarthrosis involving the tibia or fibula, with Crawford IV CPT having absolute indications for surgical treatment.

At present, there are many surgical treatment methods for Crawford IV CPT, such as vascularized free fibular transplantation, Ilizarov external fixation, IM rod fixation with bone grafting, or a combination of these technologies [2–5]. However, there is still no clear optimal plan for the effective treatment of CPT and its possible complications. The treatment difficulty of pediatric CPT has shifted from achieving initial bone healing to the treatment of various complications after bone healing [6], such as re fractures, unequal length of lower limbs, and developmental angular bending deformities of the lower limbs. The occurrence of any postoperative complication after achieving bone healing may lead to the recurrence of the pseudarthrosis, requiring the patient to undergo another surgery, which is a heavy blow to both the patient and their parents. Since 2006, we have been using combined surgery, including removal of tibial pseudarthrosis and surrounding hamartomatous tissue, internal fixation of the tibial IM rod across the ankle, wrapped autogenous iliac bone grafting, and Ilizarov external fixation, to treat Crawford IV pediatric CPT, and have achieved relatively mature and successful experience.

We retrospectively collected clinical data from 82 children with Crawford IV CPT who underwent combined surgery with tibial IM rod fixation across the ankle to answer the following questions through at least 7 years of postoperative follow-up: (1) What is the initial bone healing rate and postoperative complications of combined surgery with tibial IM rod fixation across the ankle for the treatment of Crawford IV CPT? (2) What are the technical advantages of combined surgery with tibial IM rod fixation across the ankle for the treatment of Crawford IV CPT? (3) What are the influencing factors of re fractures after initial bone healing in Crawford IV CPT treated with combined surgery with IM rod fixation across the ankle?

## Methods

### Study design and setting

This was a single-center, retrospective case series evaluating the results of combined surgery with tibial IM rod fixation across the ankle for the treatment of Crawford IV CPT. The study was conducted at urban Children’s hospital from January 2007 to December 2016.

### Patient selection

Inclusion criteria: (1) Admitted diagnosis as Crawford IV CPT in children; (2) Combined surgical treatment of tibial IM rod fixation across the ankle; (3) Follow-up time greater than 7 years after healing of tibial pseudarthrosis. Exclusion criteria: (1) Combined surgical treatment with tibial expandable IM rod fixation or other internal fixation treatments; (2) The follow-up data is incomplete.

According to the inclusion and exclusion criteria, a total of 82 Crawford IV pediatric CPT patients were included, including 47 males and 35 females, 36 cases on the right side and 46 cases on the left side; Comorbidities: 16 cases of fibular pseudarthrosis and 50 cases of neurofibromatosis type 1 (NF1). The 5 patients had a history of surgery in other hospitals and all failed.

### Surgical technique

**Fig. 1:**
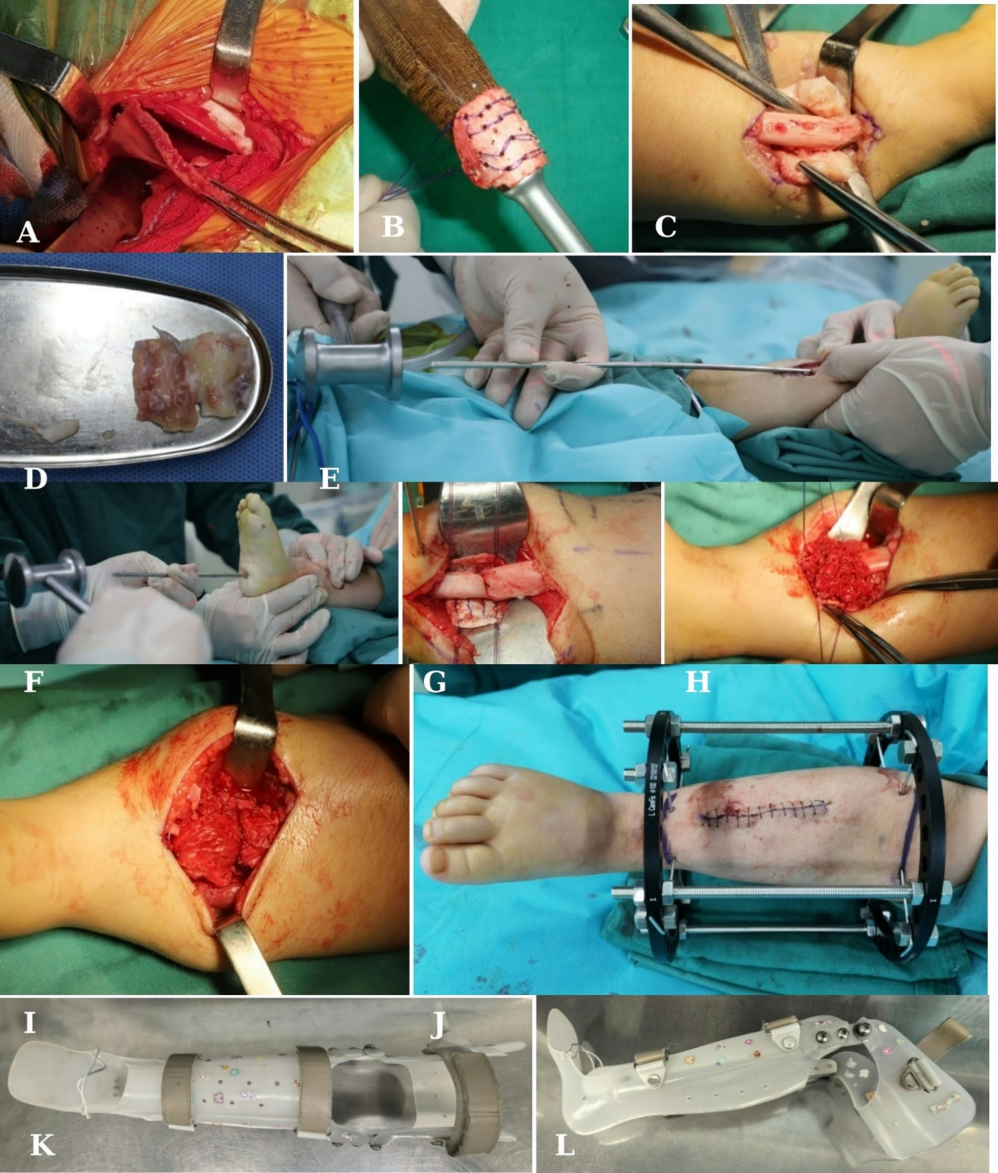
The operational steps during the combined surgery are as follows: **A**, **B** represent obtaining autologous iliac bone, creating a semi-cylindrical cortical bone shell, and weaving bone blocks with absorbable thread to prevent fracture splitting, facilitating wrapping, binding, and bone grafting; **C**, **D** represent Complete resection of the tibial pseudarthrosis and its surrounding hamartomatous lesions; **E**, **F** represent the insertion of Williams IM rods into the tibia to maintain the tibial force line; **G**, **H**, **I** represent wrapped bone grafting. The posterior and bilateral ends of the tibia are wrapped with semi-cylindrical cortical bone, and the gaps are compressed and filled with autologous cancellous bone debris. The anterior part is covered with absorbable anti adhesion film, forming a physical isolation barrier between the muscle layer and soft tissue; **J** represents the installation of Ilizarov external fixator. Preventing rotation between tibial fracture ends and maintaining compressive stress between fracture ends is beneficial for osteogenic healing; **K**, **L** represents the knee-ankle-foot brace

All the 82 patients underwent combined surgery with tibial IM rod fixation across the ankle. The brief surgical steps were as follows (As shown in Fig. 1):

*(1) Obtaining autologous iliac bone.*

A rectangular cortical bone was cut from the outer plate of the ilium, and its size should be taken as much as possible according to the donor site of the patient. As much cancellous bone as possible should be scraped and cut into small particles with bone scissors to facilitate placement in the space surrounding the bone graft. At least 4 rows of holes are drilled into the rectangular cortical bone with a 1.5 mm diameter Kirschner wire, and absorbable sutures were used to weave each hole to prevent fractures when the rectangular cortical bone was bent into a semi cylindrical shape.

*(2) Completing resection of the tibial pseudoarthrosis and surrounding hamartoma like lesions*,* and inserting Williams IM rod through the ankle.*

The tibial pseudoarthrosis and its surrounding hamartoma like lesions must be carefully and completely resected like tumor tissue, with minimal residual lesion tissue. After the diseased tissue between the broken ends of the tibia was completely bitten off, a moderate alignment was maintained between the broken ends. The insertion positions of IM rods in the proximal and distal segments of the tibial pseudoarthrosis were located under fluoroscopy using a 2.0 mm diameter Kirschner wire, keeping the positioning needle perpendicular to the distal and proximal epiphyseal lines of the tibia and located in the center of the tibial medullary cavity. According to the diameter of the implanted IM rod, the proximal and distal medullary cavities of the tibial pseudoarthrosis should be expanded along the direction of the positioning needle using appropriately sized hollow drills, especially in areas of osteosclerosis where the medullary cavity must be opened. The appropriate Williams IM rod was selected based on the length and diameter of the tibia measured on the patient’s preoperative X-ray film. The IM rod was inserted from the proximal end of the distal segment of the tibial pseudoarthrosis to the distal end, passing through the distal tibial epiphyseal plate, talus, and calcaneus, and penetrating the plantar area of the foot from the heel pad, maintaining a neutral position of the ankle in the sagittal and coronal positions. Then, the IM rod was retrograde inserted into the proximal medullary cavity of the tibial pseudoarthrosis until it reached the plane of the proximal tibial epiphyseal plate, keeping the distal and proximal ends of the tibial pseudoarthrosis tightly attached and maintaining a certain pressure to avoid torsion of the distal and proximal segments of the tibial pseudoarthrosis.

*(3) Wrapped autologous iliac bone transplantation.*

After inserting the IM rod, the wound was rinsed and stopped bleeding. The area around the broken ends of the tibial pseudoarthrosis (mainly the posterior and bilateral sides) was surrounded by previously obtained semi cylindrical iliac cortical bone, and the gaps between the broken ends and the wrapped cortical bone were filled with cancellous bone to keep the cancellous bone tightly packed without leaving any gaps. The bone graft site was bound and fixed with absorbable sutures, and its front was covered with an absorbable anti adhesion film (DIKANG BIOMEDICAL CO.,LTD) to provide physical isolation between the muscle layer and the bone graft site, preventing the residual hamartoma like lesion tissue in the muscle layer soft tissue from invading again before the bone graft site was fully osteogenic and healed. Then, the bone graft site was covered with the anterior lateral calf muscle group, and the incision was sequentially sutured layer by layer.

*(4) Installing the Ilizarov external fixator.*

According to the preoperative design, a complete ring was separately installed at the level of the distal and proximal epiphyseal plates of the tibia, and two to three 1.5–2.0 mm Kirschner wires were used to cross fix the rings. The lower leg was kept in the center of the rings, and four connecting rods were used to connect these two rings to maintain a certain pressure between the two rings and compress the broken ends, avoiding torsion and separation between the distal and proximal segments of the tibia.

### Postoperative protocol

After surgery, regular follow-up X-ray films were taken to observe the healing of tibial pseudarthrosis and the occurrence of complications. If it was confirmed that the bone trabeculae at both ends of the pseudoarthrosis had been bridged, the Ilizarov external fixation device could be removed and replaced with a long leg cast for 2 months. When the both ends of the tibial pseudoarthrosis were confirmed to be firmly healed by rechecking the X-ray film, a knee-ankle-foot brace (As shown in Fig. 1) could be customized to protect the affected limb. Patients should wear braces when standing or moving on the ground, and try to avoid accidents such as injuries. they were allowed to walk under the protection of braces and engage in general sports activities, severe sports activities were strictly prohibited. After one year of bone healing, some patients chose to push the IM rod into the tibia mainly to release the long-term fixation of the ankle, hoping to improve the function of the ankle. Some patients chose IM rod in situ fixation mainly due to poor healing of the tibial pseudarthrosis or incomplete healing of the tibial pseudarthrosis. Some patients chose to remove the IM rod mainly because the tibial pseudarthrosis had already achieved complete healing.

### Follow-up methods and observation indicators

All patients will undergo bilateral tibiofibular anteroposterior and lateral radiographs, as well as bilateral lower limb full-length radiographs at 1, 3, 6, and 12 months postoperatively. Follow-ups will be conducted annually thereafter, with regular follow-up until skeletal development was mature.

1. Evaluation of tibial pseudarthrosis healing: According to the Radiographic Union Score for Tibial Fractures (RUST) [7], those with a score of ≥ 8 are considered as initial bone healing of the tibial pseudarthrosis, while those with a score of < 8 are considered as delayed or non-healing of the tibial pseudarthrosis.
2. Judgment of re fracture after bone healing: If the X-ray film shows that there is cortical discontinuity, negative shadow of the fracture line, or misaligned angle again after the healing of the tibial pseudarthrosis, it is considered to be a re fracture.
3. Diagnosis of developmental angular curvature deformity of the tibial shaft: Picture archiving and communication systems (PACS) are used on the X-ray films to measure whether there are angular deformities in the distal and proximal anatomical axes of the affected tibia in the sagittal and/or coronal planes.
4. Measurement of proximal tibial valgus angle: The PACS are used to measure the angle between the parallel epiphyseal plate line of the proximal tibia and the anatomical axis of the upper tibia. If the angle is greater than 3°, it is defined as proximal tibial valgus.
5. Measurement of ankle valgus angle: The PACS are used to measure the angle between the anatomical axis of the lower segment of the tibia and the tibial talar joint surface. If the angle is greater than 5°, it is defined as ankle valgus.
6. Measurement of unequal length of lower limbs: The PACS are used to measure the length of both femur and tibia, that is, the distance between the midpoint of the proximal femoral plates and the midpoint of the distal femoral plates, and the distance between the midpoint of the proximal tibial plates and the midpoint of the distal tibial plates.
7. Diagnosis of tibial IM rod displacement: After the tibial IM rod naturally retreats or is pushed and retained in the tibial medullary cavity with the growth and development of the tibia, the tibial IM rod that lacks stable fixation deviates from the central position of the medullary cavity due to long-term repeated movements of the affected limb.

### Ethical approval

This study has been approved by the Ethics Committee of our Hospital, and all legal guardians of the patients have obtained informed consent and signed an informed consent form.

### Statistical analyses

The data was organized and analyzed using SPSS 19.0 statistical software. Measurement data represented in x ®±s, independent sample t-test was used for inter group comparison; The count data was expressed in terms of examples and percentages, and chi-square tests were used for inter group comparisons. The difference was statistically significant with *P* < 0.05.

## Results

### Basic information and bone union status after CPT surgery

All patients were followed up for an average of (119.78 ± 20.08) months (range: 84–146 months) postoperatively, with an average surgical age of (43.17 ± 13.40) months (range: 18–96 months). All patients achieved initial bone healing of the tibial pseudarthrosis, with an initial bone healing rate of 100% and an average initial bone healing time of (4.16 ± 0.65) months (range: 3–6 months). At the last follow-up, there were 25 cases with mature skeletal development and 57 cases with immature skeletal development.

### The relationship between the fixation status of tibial IM rods and the displacement of tibial IM rods, developmental tibial shaft angular bending deformity, and re fractures after the healing of the tibial pseudarthrosis

After the healing of the tibial pseudarthrosis, the patients were divided into three groups based on the fixation status of the tibial IM rod: the complete insertion of IM rods into the tibial medullary cavity group (37 cases), the tibial IM rod across the ankle fixation group (25 cases), and the tibial IM rod removal group (20 cases). There was no statistically significant difference in follow-up time among the three groups of cases.

At the last follow-up, all cases in the complete insertion of IM rods into the tibial medullary cavity group showed tibial IM rods deviating from the center of the medullary cavity, with a displacement rate of up to 100%, which significantly affected the normal development of the tibia, leading to the tibia being cut by the IM rod and causing tibia re fracture. Among them, 10 cases protruded from the tibial cortex and underwent IM rod removal; All cases in the tibial IM rod across the ankle fixation group showed no displacement of the tibial IM rod.

At the last follow-up, there were a total of 20 cases of re fractures, and 16 children with a history of trauma. After a series of treatments, bone healing was achieved again. Among them, there were 15 cases in the complete insertion of IM rods into the tibial medullary cavity group, 2 cases in the tibial IM rod across the ankle fixation group, and 3 cases in the tibial IM rod removal group. The differences between the groups were statistically significant (χ^2^ = 9.831, *P* = 0.007).

At the last follow-up, there were a total of 28 cases of developmental bending and angular deformity of the tibial shaft after bone healing, including 26 cases in the complete insertion of IM rods into the tibial medullary cavity group, 0 cases in the tibial IM rod across the ankle fixation group, and 2 cases in the tibial IM rod removal group. The differences between the groups were statistically significant (χ^2^ = 39.620, *P* = 0.000).

**Fig. 2:**
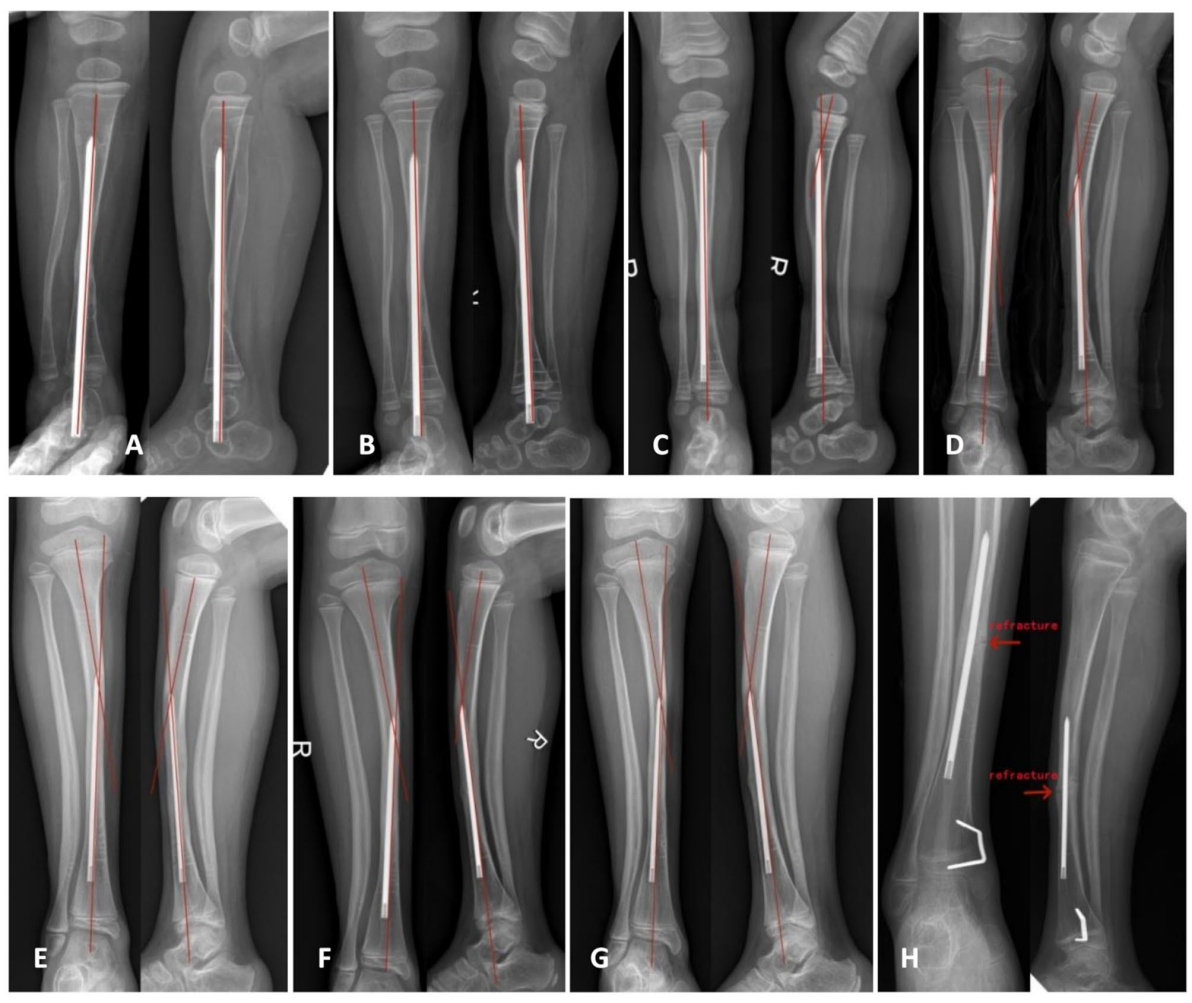
A case of tibial pseudarthrosis treated with combined surgery followed up for more than 10 years for X-ray changes after bone healing. It can be seen that the angular deformity of the tibial shaft gradually forms and worsens in the sagittal and coronal planes, accompanied by displacement of the tibial intramedullary rod and re fracture. **A** represented the anteroposterior and lateral views at six months after bone healing; **B** represented the anteroposterior and lateral views at 1 year after bone healing; **C** represented the anteroposterior and lateral views at one and a half years after bone healing, showing angular deformity of the tibial shaft in the sagittal plane; **D** represented the anteroposterior and lateral views at 4 years after bone healing, showing angular deformity of the tibial shaft in the sagittal and coronal planes; **E** represented the anteroposterior and lateral views at 6 years after bone healing, showing angular deformity of the tibial shaft in the sagittal and coronal planes; **F** represented the anteroposterior and lateral views at 7 years after bone healing; **G** represented the anteroposterior and lateral views at 8 years after bone healing; **H** represented the anteroposterior and lateral views at 10 and a half years after bone healing, showing angular deformities of the tibial shaft in the sagittal and coronal planes, accompanied by displacement of the tibial IM rod and re fracture

In Figs. 2, a typical patient with tibial pseudarthrosis treated with combined surgery of tibial IM rod fixation across the ankle was presented, demonstrating the occurrence and development of complications such as IM rod displacement, developmental tibial shaft angular bending deformity, and re fracture after the IM rod was fully inserted into the tibia.

Distribution of developmental angular deformities and re fractures of the tibial shaft after bone healing at different follow-up periods under different fixation states of tibial IM rods were shown in Tables 1, 2 and 3. 70% of re fractures occurred more than 3 years after bone healing; 82% of developmental angular deformities of the tibial shaft occurred more than 3 years after bone healing.

**Table 1:** The incidence of developmental angular deformities and re fractures of the tibial shaft under different fixation states of tibial IM rods at the last follow-up

fixation states of IM rods	sum	angular deformities of tibial shaft	re fractures
IM rod insertion group [n(%)]	37(45.1)	26(70.3)	15(40.5)
IM rod in situ fixation group[n(%)]	25(30.5)	0(0.0)	2(8.0)
IM rod removal group[n(%)]	20(24.4)	2(10.0)	3(15.0)
*P*-value		0.000	0.007

Note: The calculation of *P*-value adopts the χ^2^ test

**Table 2:** Distribution of developmental angular deformities after bone healing of the tibial shaft under different fixed states during different follow-up periods

fixation states of IM rods	follow-up times				sum	*P*-value
	≤ 1 year	1 ~ 3 years	3 ~ 6 years	≥ 6 years		
IM rod insertion group	0	5	10	11	26	
IM rod in situ fixation group	0	0	0	0	0	0.000
IM rod removal group	0	0	1	1	2	

Note: The calculation of *P*-value adopts the χ^2^ test

**Table 3:** Distribution of re fractures after bone healing of the tibial shaft under different fixed states during different follow-up periods

fixation states of IM rods	follow-up times				sum	*P*-value
	≤ 1 year	1 ~ 3 years	3 ~ 6 years	≥ 6 years		
IM rod insertion group	1	2	6	6	15	
IM rod in situ fixation group	1	1	0	0	2	0.007
IM rod removal group	0	1	1	1	3	

Note: The calculation of *P*-value adopts the χ^2^ test

### Other complications after CPT surgery

There were 10 cases of proximal tibial valgus, all of which were treated with “8” - shaped plate. There were 12 cases of ankle valgus, of which 10 were treated with U-shaped nails, and 2 severe cases that could not be corrected through semi epiphyseal block underwent ankle osteotomy for correction. There were 15 cases of unequal length of the lower limbs, including 5 cases with longer tibia on the affected side and 10 cases with longer tibia on the healthy side and compensatory overgrowth of the affected femur (ranging from 1 to 2 centimeters). The average length difference between the lower limbs was (2.71 ± 1.39) cm, with a range of (1.1 to 5.0) cm. Among them, 5 cases had a length difference of less than 2 cm and were treated with elevated insoles; Six cases with a length difference greater than 4 cm were successfully treated with proximal tibiofibular lengthening on the shorter limb; Four cases with a length difference of 2–4 cm were treated with total epiphyseal block at the proximal tibial metaphysis on the longer limb.

There were 9 cases of distal tibial epiphyseal plate bone bridge formation, 10 cases of Ilizarov external fixation needle infection, 2 cases of tibial osteomyelitis, 35 cases of ankle stiffness, and 16 cases of persistent fibular pseudarthrosis after the tibial pseudarthrosis healing.

## Discussion

### Current status of surgical treatment for Crawford IV CPT

The treatment of pediatric Crawford IV CPT is extremely challenging for pediatric orthopedic physicians [4, 8], and the main goal of surgical treatment is to achieve long-term bone healing of the tibia, prevent re fractures after bone healing, unequal length of the lower limbs, and postoperative tibial mechanical axis angular deformity. The main contents of the surgery include resection of the tibial pseudarthrosis and its surrounding hamartomatous tissue, stable fixation between tibial fracture ends, and bone grafting to promote tibial fracture healing or repair bone defects. The different surgical treatment methods mainly manifest in the different fixation methods and bone grafting methods between the tibial fracture ends. The most commonly used surgical fixation methods currently include tibial IM rod fixation, Ilizarov external fixation, and a combination of IM rod fixation and Ilizarov external fixation [2, 4] and so on; The commonly used bone grafting methods include double-layer cortical or segmental bone grafting [9], iliac cortical and cancellous bone transplantation [10], tibiofibular cross fusion bone grafting [11–16] (such as “three in one” or “four in one” surgery), and vascular free fibular transplantation [17], etc. The commonly used types of bone grafting include allogeneic or artificial bone transplantation, as well as autologous bone transplantation or mixed autologous and allogeneic bone transplantation. Other adjuvant treatment methods mainly include improving biological characteristics by using bone morphogenetic protein (BMP) or preoperative use of bisphosphonates to promote bone healing between tibial fractures. In recent years, with the continuous improvement of surgical methods, the initial bone healing rate after surgery has also been continuously improved. Research analysis [18] had shown that the combined technique of IM rod fixation with Ilizarov external fixator and cortical bone transplantation had the best treatment results in terms of healing time, bone healing quality, and reducing the number of fractures. The combination of IM rod fixation and Ilizarov external fixation had been more widely used than any other single fixation method, because this combined fixation could maintain the stability of the tibial mechanical axial force line through IM rod fixation, as well as increase the longitudinal pressure applied to the fractured end of the pseudarthrosis and the axial anti torsion force of the tibia through Ilizarov external fixation, which was currently considered one of the best stable fixation methods for treating CPT. Although IM rod fixation across the ankle could damage the ankle and lead to postoperative complications such as ankle stiffness, its ability to maintain the axis of the tibia and stability of fixation was significantly enhanced, which was more conducive to osteogenic healing between the broken ends of the tibia. With the improvement of technology, extendable IM rods have gradually been widely used. As the tibia grows, extendable IM rods can slide and extend, maintaining the mechanical axis of the tibia for a long time and providing continuous support to the tibia, thereby reducing the occurrence of postoperative fractures; At the same time, its fixation does not pass through the ankle, which can avoid damage to ankle function. However, the installation of extendable IM rods during surgery required insertion through the front of the proximal tibial plateau, which could damage the proximal tibial epiphysis and cause bone bridge formation; At the same time, the expandable IM rod was designed with a hollow structure, which had poor fixation strength and stability. The intraoperative technical requirements were high, and it was prone to postoperative sliding failure, which could cause the distal and proximal fixation threads of the expandable IM rod to slip from the epiphysis, ultimately leading to displacement of the IM rod and the occurrence of re fractures; In addition, the distal thread of the expandable IM rod need to be screwed into the epiphyseal plate of the distal tibia for fixation, which could also cause damage or tethering to the distal tibial epiphyseal plate. Although Paley et al. [19] reported the use of extendable IM rods in cross fusion technology, which avoided damage to ankle function, and achieved an initial bone healing rate of 100% with no postoperative fractures, the long-term follow-up results are still worth observing, and further follow-up is needed for postoperative complications. Moreover, in Paley’s cross fusion technique, bone grafts were entirely made using autologous iliac cancellous bone, which required a large amount of cancellous bone during surgery. This technique might not be suitable for younger children or those with poor pelvic iliac bone development. In this study, we used the combined technique of Williams IM rod fixation across the foot and ankle to treat CPT, which is currently the largest single center case series report. The research results showed that the initial bone healing rate reached 100%, with no amputation in children.

### Technical advantages and theoretical basis of combined surgery with tibial IM rod fixation across the ankle for the treatment of Crawford IV congenital tibial pseudarthrosis

Our combined surgery included complete resection of the tibial pseudarthrosis and surrounding hamartomatous tissue, wrapped autogenous iliac bone grafting, and Williams IM rod combined with Ilizarov external fixator fixation across the ankle. The combination of these three measures is the greatest advantage of our combined surgical technique, and each measure is aimed at the characteristics of the disease that is difficult to heal, which can significantly improve the initial bone healing rate after surgery, The surgery has a wide range of applications and can be applied to simple Crawford IV pediatric CPT, especially for cases of single or multiple surgical failures. The results of this study indicated that our high initial bone healing rate was mainly related to our unique wrapped tight bone grafting method and the combined fixation of Williams IM rod across the ankle and Ilizarov external fixator. We removed the tibial pseudarthrosis and its surrounding hamartoma like tissue as completely as possible in the combined surgery, reducing the local invasiveness of the hamartoma like tissue. At the same time, the bone graft site was wrapped with semi-cylindrical bone cortex around the broken ends of the tibial pseudarthrosis (mainly the posterior and bilateral), and the cancellous bone tightly filled the bone graft gap, And in the area lacking cortical wrapping at the front of the semi-cylindrical bone shell, an absorbable anti adhesion film was used to isolate the cancellous bone grafting site from the surrounding soft tissue, forming a cylindrical physical isolation barrier, further preventing the re invasion of hamartoma like lesion tissue that had not been completely removed in the soft tissue around the bone grafting site before osteogenic healing, thereby ensuring a favorable microenvironment for osteogenesis at the bone grafting site. Paley et al. [20] believed that the treatment of pediatric CPT must fully consider the biological and biomechanical pathophysiological characteristics of the tibial pseudarthrosis. Shah et al. [21] pointed out in a multicenter long-term follow-up study on the influencing factors of bone healing after CPT surgery that the selected bone graft type and the factors of ankle and subtalar joint fixation were important factors affecting early bone healing. In the combined surgery, we fully recognized the biological and biomechanical pathophysiological characteristics of the tibial pseudarthrosis, completely removed the invasive hamartoma tissue, used the characteristic bone grafting method of rearrangement of cortical and cancellous bone of autogenous iliac bone, and selected the combined fixation method of Williams intramedullary rod across the ankle and Ilizarov external fixation frame. The combined application of the three measures significantly improved the early postoperative bone healing rate.

### The influencing factors of re fractures after initial bone healing in Crawford IV CPT treated with combined surgery with IM rod fixation across the ankle

The re fracture after initial bone healing was a serious complication after surgical treatment of CPT. The causes of re fracture after bone healing were relatively complex. Currently, there are few reports on the relevant risk factors for re fracture after initial bone healing of pseudarthrosis both domestically and internationally, and most of them are small-sample univariate studies. After long-term follow-up studies in this study, we believe that the re fracture after bone healing may be the result of a combination of the following risk factors: ①The strength of local bone after the healing of tibial pseudarthrosis. It was mainly related to the cross-sectional area of bone healing and the osteogenic quality of local cortical and cancellous bone. The stronger the local bone strength after the healing of tibial pseudarthrosis, the lower the chance of re fracture. The cross fusion technique proposed by Paley et al. [19] was precisely because it relatively increased the cross-sectional area of the bone healing site, thereby enhancing the local bone strength at the healing site and reducing the risk of re fracture after bone healing. Deng Huajun et al. [22] believed that the larger the cross-sectional area of the bone healing site, the longer the fracture free survival time. During surgery, increasing the cross-sectional area of the bone healing site as much as possible could reduce the risk of re fracture after bone healing; ②The state of the mechanical axis of the tibia. The long-term follow-up results of this study showed that developmental angular bending of the tibial shaft was more likely to lead to postoperative re fractures; ③External influencing factors for re fracture after bone healing, such as the presence of a history of trauma. The long-term follow-up results of this study showed that 80% of cases of re fractures after bone healing had a history of trauma. Even normal bone could cause fractures after trauma, so the occurrence of re fractures after bone healing couldn’t be ruled out by the influence of traumatic factors; ④The state of the fibula after healing of the tibial pseudarthrosis. The integrity of the fibula was directly related to the sharing of weight-bearing pressure on the tibia of the affected limb. A complete fibula could reduce the weight-bearing pressure on the tibia. Therefore, the integrity of the fibula was a related factor in the occurrence of re fractures after bone healing. Liu Yaoxi et al. [23] found that the incidence of re fractures in patients with CPT combined with incomplete fibula was higher than that in patients with CPT combined with intact fibula, and there was a statistically significant difference. Deng Huajun et al. [24] found that the average fracture free survival time of CPT with fibular pseudarthrosis was shorter than that of CPT with intact fibula, and CPT patients with fibular pseudarthrosis were more prone to re fractures; ⑤The fixation status of the tibial IM rod. The long-term follow-up results of this study showed that the fixation status of the tibial IM rod after bone healing significantly affected the long-term development of the tibia shaft. The incidence of IM rod displacement after being fully inserted and retained in the tibia is as high as 100%. The incidence of developmental angular bending deformity and re fracture of the tibial shaft in the group with complete insertion of the IM rod into the tibial medullary cavity is significantly higher than that in the group with the tibial IM rod across the ankle fixation and removal of the IM rod, and there is a statistically significant difference in incidence between the groups (*P* < 0.05), indicating that the combined surgical treatment of IM rod fixation across the ankle can cause developmental angular bending deformity of the tibial shaft and increases the risk of re fractures when the tibial IM rod is fully inserted and left in the tibial medullary cavity after bone healing. We speculate that this may be due to the long-term lack of stable fixation of the IM rod in the tibial medullary cavity, which causes uneven stress after repeated movements of the lower limbs, leading to chronic cutting of the tibial bone, affecting the normal development of the tibial bone, weakening the local bone strength of the tibia, and ultimately leading to displacement of the IM rod and developmental angular bending deformity of the tibial shaft. Under external forces, it may lead to re fracture of the tibial shaft. Therefore, we believe that the fixation status of tibial IM rods is an important influencing factor for re fractures after initial bone healing, after the healing of the pseudarthrosis, the tibial IM rod should not be fully inserted into the tibial medullary cavity to release the fixation of the ankle, which can easily lead to displacement of the tibial IM rod, increase the risk of tibial shaft developmental angular bending deformity and re fracture after the bone healing.

### Other complications after CPT surgery

In addition, the long-term follow-up results of this study indicate that after combined surgical treatment, complications such as unequal length of the lower limbs, proximal tibial valgus, ankle valgus, distal tibial epiphyseal plate bone bridge, Ilizarov external fixator needle infection, tibial osteomyelitis, and ankle joint stiffness can also occur, and timely and symptomatic treatment is necessary to avoid recurrence of tibial pseudarthrosis.

### Limitations

This study has certain limitations. Firstly, this article is a retrospective study, which inevitably leads to selective bias; Secondly, the postoperative follow-up time of the patients is not long enough, and some patients have not been followed up until their bones are mature, making it difficult to accurately calculate the incidence of postoperative complications. At the same time, the number of samples is not large enough and the conclusions drawn need more prospective studies or multicenter studies to increase the sample size, further confirming our conclusions; Thirdly, this study lacks alternative methods as a control group to compare the therapeutic effects of different treatment methods, and the therapeutic effects are not convincing, and there is no biomechanical laboratory study to compare different fixation methods under different conditions. However, these limitations do not appear to undermine the results achieved in this study. It is strongly recommended to further study different variables such as patient’s age, etiology and severity of malformation and functional results. Fourthly, our research only represents the experience of one institution and requires extensive promotion and application by multiple institutions and centers for research validation.

## Conclusions

In summary, the combined surgery of tibial IM rod fixation across the ankle for the treatment of Crawford IV CPT has a high initial bone healing rate and definite therapeutic effect, but there are still many postoperative complications. The fixation status of tibial IM rods is an important influencing factor for tibial shaft developmental angular bending deformity and re fractures after initial bone healing, after the healing of the pseudarthrosis, the tibial IM rod should not be fully inserted into the tibial medullary cavity to release the fixation of the ankle, which can easily lead to displacement of the tibial IM rod, increase the risk of tibial shaft developmental angular bending deformity and re fracture after the bone healing.

## Data Availability

Data are available by request.

## Abbreviations

BMP: Bone Morphogenetic Protein
CPT: Congenital pseudarthrosis of tibia
IM: Intramedullary
NF-1: Neurofibromatosis type 1
PACS: Picture archiving and communication systems
RUST: The Radiographic Union Score for Tibial Fractures

## References

[1] Hefti F, Bollini G, Dungl P, et al. Congenital pseudarthrosis of the tibia: history, etiology, classification, and epidemiologic data. J Pediatr Orthop B. 2000;9:11–5. 10647103 10.1097/01202412-200001000-00003

[2] Agashe MV, Song SH, Refai MA, et al. Congenital pseudarthrosis of the tibia treated with a combination of ilizarov’s technique and intramedullary rodding. Acta Orthop. 2012;83:515–22. 23043268 10.3109/17453674.2012.736170 PMC3488180

[3] Haibo M, Rongguo H, Kun L, et al. Intramedullary rod combined with ring external fixation for treatment of congenital pseudarthrosis of tibia in children. Chin J Pediatr Surg. 2012;33(6):421–5.

[4] Khan T, Joseph B. Controversies in the management of congenital pseudarthrosis of the tibia and fibula. Bone Joint J. 2013;95:1027–34. 23908415 10.1302/0301-620X.95B8.31434

[5] Paley D. In: Zorzi A, editor. Congenital pseudarthrosis of the tibia: combined Pharmacologic and surgical treatment using biphosphonate intravenous infusion and bone morphogenic protein with periosteal and cancellous autogenous bone grafting, tibio-fibular cross union, intramedullary rodding and external fixation. Reijeca, Croatia: InTech; 2012. pp. 91–106.

[6] Inan M, El Rassi G, Riddle EC, et al. Residual deformities following successful initial bone union in congenital pseudoarthrosis of the tibia. J Pediatr Orthop. 2006;26(3):393–9. 16670555 10.1097/01.bpo.0000217716.64986.f0

[7] Richards BS, Wilkes D, Dempsey M, et al. A radiographic scoring system to assess healing in congenital pseudarthrosis of the tibia. J Pediatr Orthop B. 2015;24(2):118–22. 25588045 10.1097/BPB.0000000000000141

[8] Pannier S. Congenital pseudarthrosis of the tibia. Orthop Traumatol Surg Res. 2011;97(7):750–61. 21996526 10.1016/j.otsr.2011.09.001

[9] Shah H, Doddabasappa SN, Joseph B. Congenital pseudarthrosis of the tibia treated with intramedullary rodding and cortical bone grafting: a follow-up study at skeletal maturity. J Pediatr Orthop. 2011;31(1):79–88. 21150736 10.1097/BPO.0b013e318202c45d

[10] Haibo M, Rongguo H, Kun L, et al. Effect of enclosing autogenous iliac bone graft in treatment for congenital pseudarthrosis of the tibial in children. J Clin Pediatr Surg. 2011;10(3):163–6.

[11] Chunxing W, Guizhou Z, Dahui W, et al. Combination treatment by cross fusion of the tibia and fibula, autogenic iliac bone grafting, reliable fixation and bone morphogenetic proteins for the treatment of refractory congenital pseudarthrosis of the tibia. J Pediatr Orthop. 2022;42:e623–9. 35297391 10.1097/BPO.0000000000002138 PMC9165645

[12] Choi IH, Lee SJ, Moon HJ, et al. 4-in-1 osteosynthesis for atrophic-type congenital pseudarthrosis of the tibia. J Pediatr Orthop. 2011;31(6):697–704. 21841448 10.1097/BPO.0b013e318221ebce

[13] Jiangyan W, Haibo M, Yaoxi L, et al. Application of 3-in-1 osteosynthesis in combined surgical technique for congenital pseudarthrosis of the tibia in children. Chin J Pediatr Surg. 2017;38(9):691–7.

[14] Liu Y, Yang G, Liu K, et al. Combined surgery with 3-in-1 osteosynthesis in congenital pseudarthrosis of the tibia with intact fibula. Orphanet J Rare Dis. 2020;15:62. 32122367 10.1186/s13023-020-1330-z PMC7053109

[15] Shannon CE, Huser AJ, Paley D. Cross-union surgery for congenital pseudarthrosis of the tibia. Children. 2021;8:547. 34202921 10.3390/children8070547 PMC8303361

[16] Yaoxi L, Haibo M, Kun L, et al. Application of 4-in-1 osteosynthesis in the combined surgical technique for the treatment of congenital pseudarthrosis of tibia in children. Chin J Orthop. 2016;36(12):770–7.

[17] Vigouroux F, Mezzadri G, Parot R, et al. Vascularised fibula or induced membrane to treat congenital pseudarthrosis of the tibia: a multicentre study of 18 patients with a mean 9.5-year follow-up. Orthop Traumatol Surg Res. 2017;103(5):747–53. 28559144 10.1016/j.otsr.2017.05.005

[18] Kesireddy N, Kheireldin RK, Lu A, et al. Current treatment of congenital pseudarthrosis of the tibia: a systematic review and meta-analysis. J Pediatr Orthop B. 2018;27:541–50. 29878977 10.1097/BPB.0000000000000524

[19] Paley D. Paley cross-union protocol for treatment of congenital pseudarthrosis of the tibia. Oper Tech Orthop. 2021;31(2):100881.

[20] Paley D. Congenital pseudarthrosis of the tibia: biological and biomechanical considerations to achieve union and prevent refracture. J Child Orthop. 2019;13(2):120–33. 30996736 10.1302/1863-2548.13.180147 PMC6442511

[21] Shah H, Joseph B, Nair BVS, et al. What factors influence union and refracture of congenital pseudarthrosis of the tibia? A multicenter long-term study. J Pediatr Orthop. 2018;38(6):e332–7. 29664876 10.1097/BPO.0000000000001172

[22] Huajun D, Haibo M, Weihua Y, et al. Combined surgery for refractures of congenital pseudarthrosis of the tibia: a report of 24 cases. Chin J Pediatr Surg. 2017;38(4):296–300.

[23] Yaoxi L, Haibo M, Guanghui Z, et al. Relationship of postoperative complications and fibula integrity in congenital pseudarthrosis of the tibia in children. Chin J Pediatr Surg. 2016;37(8):573–6.

[24] Huajun D, Haibo M, Wei Z. Risk factors related to refracture after union of congenital pseudarthrosis of the tibia with a combined surgical. Chin J Orthop. 2018;38(3):164–71.

